# Bipolar forceps coagulation for endoscopic papillectomy-related bleeding

**DOI:** 10.1055/a-2440-6432

**Published:** 2024-11-08

**Authors:** Kozue Shibasaki, Haruo Miwa, Yuichi Suzuki, Kazuki Endo, Ritsuko Oishi, Hiromi Tsuchiya, Shin Maeda

**Affiliations:** 1 26437 Gastroenterological Center, Yokohama City University Medical Center, Yokohama, Japan; 2 Department of Gastroenterology, Yokohama City University Graduate School of Medicine, Yokohama, Japan

Endoscopic papillectomy is widely performed as a less invasive procedure for ampullary tumors compared with surgery 1 2 ; however, it occasionally causes refractory bleeding. Endoscopic hemostasis with clipping or electrocoagulation is used for pulsatile bleeding 3 ; however, there is a risk of perforation at the post-endoscopic papillectomy ulcer. Under these conditions, electrocoagulation using bipolar hemostatic forceps (Hemostat Y; Pentax, Tokyo, Japan) effectively minimizes the risk of excessive tissue injury 4 5 . Herein, we report two cases of endoscopic papillectomy-related bleeding that were successfully treated using bipolar forceps ( Video 1 ).

Use of bipolar hemostatic forceps was effective for pulsatile bleeding while preventing tissue injury to post-endoscopic papillectomy ulcers.Video 1

Case 1: A 61-year-old woman with an ampullary tumor underwent an endoscopic papillectomy. Pulsatile bleeding was observed at the center of the ulcer immediately after en bloc resection with a snare. First, prophylactic clipping was performed on the anal side. Subsequently, the bleeding point was grasped and coagulated by using the bipolar hemostatic forceps. No further bleeding was observed after flushing with saline solution ( Fig. 1 ). Additional clipping was performed to prevent rebleeding. Pancreatic and biliary stents were placed, and self-assembling peptide was sprayed ( Fig. 2 ). The patient was discharged on the seventh day after the endoscopic papillectomy, without any complications.

**Fig. 1:**
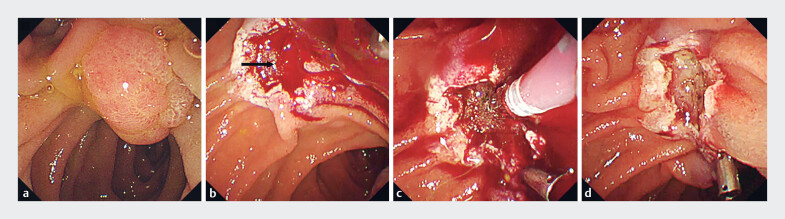
Case 1. Endoscopic papillectomy and hemostasis for bleeding. **a** An ampullary tumor located at the papilla of Vater. **b** Pulsatile bleeding was observed at the center of the post-endoscopic papillectomy ulcer (arrow). **c** After prophylactic clipping, electrocoagulation was performed using bipolar hemostatic forceps. **d** No further bleeding was observed.

**Fig. 2:**
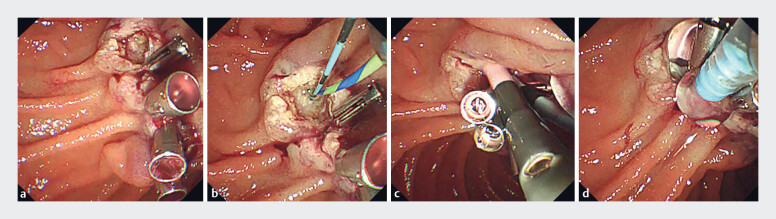
Case 1. Procedures after hemostasis. **a** Additional clipping was performed. **b** Guidewires were deployed in the bile duct and the pancreatic duct. **c** Biliary and pancreatic duct stents were placed. **d** Self-assembling peptide was sprayed to cover the post-endoscopic papillectomy ulcer.

Case 2: A 51-year-old woman with an ampullary tumor underwent endoscopic papillectomy ( Fig. 3 ). The following day, she experienced hematemesis, and emergency endoscopy was performed. The post-endoscopic papillectomy ulcer was covered with clots and fresh blood. Pulsatile bleeding was detected around the pancreatic stent after clot removal and flushing with saline solution. The bipolar hemostatic forceps was used to accurately grasp the bleeding point. Endoscopic hemostasis was successfully achieved after two instances of bipolar coagulation. No further bleeding was observed, and the patient was discharged as scheduled ( Fig. 4 ).

**Fig. 3:**
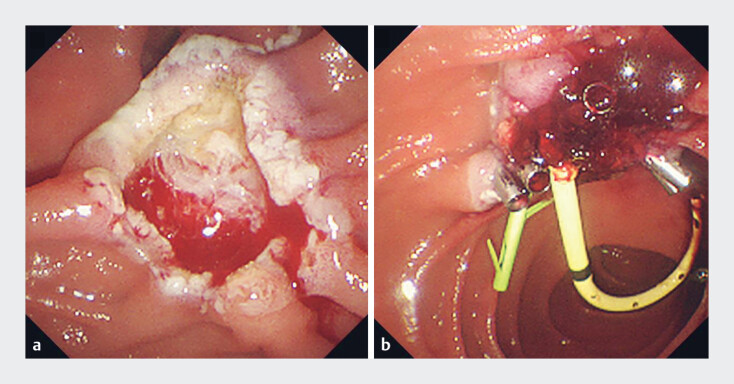
Case 2. Endoscopic papillectomy. **a** Oozing was observed during the procedure. **b** The bleeding spontaneously ceased, and prophylactic clipping was performed.

**Fig. 4:**
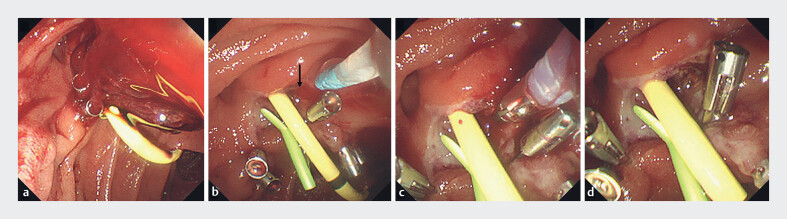
Case 2. Emergency endoscopy on the following day. **a** The post-endoscopic papillectomy ulcer was covered with clots and fresh blood. **b** Pulsatile bleeding was detected around the pancreatic stent (arrow). **c** Coagulation hemostasis was achieved using bipolar hemostatic forceps. **d** Self-assembling peptide was sprayed, and no further bleeding was observed.

To the best of our knowledge, this is the first report of endoscopic hemostasis using bipolar forceps coagulation for endoscopic papillectomy-related bleeding. This device is effective for pulsatile bleeding while preventing tissue injury to post-endoscopic papillectomy ulcers.

Endoscopy_UCTN_Code_CPL_1AK_2AC

## Notes

Endoscopy E-Videos https://eref.thieme.de/e-videos
*E-Videos* is an open access online section of the journal *Endoscopy* , reporting on interesting cases and new techniques in gastroenterological endoscopy. All papers include a high-quality video and are published with a Creative Commons CC-BY license. Endoscopy E-Videos qualify for HINARI discounts and waivers and eligibility is automatically checked during the submission process. We grant 100% waivers to articles whose corresponding authors are based in Group A countries and 50% waivers to those who are based in Group B countries as classified by Research4Life (see: https://www.research4life.org/access/eligibility/ ). This section has its own submission website at https://mc.manuscriptcentral.com/e-videos .
